# Ten_m3 Regulates Eye-Specific Patterning in the Mammalian Visual Pathway and Is Required for Binocular Vision 

**DOI:** 10.1371/journal.pbio.0050241

**Published:** 2007-09-04

**Authors:** Catherine A Leamey, Sam Merlin, Paul Lattouf, Atomu Sawatari, Xiaohong Zhou, Natasha Demel, Kelly A Glendining, Toshitaka Oohashi, Mriganka Sur, Reinhard Fässler

**Affiliations:** 1 Department of Physiology, Bosch Institute and School of Medical Sciences, University of Sydney, Sydney, Australia; 2 Brain and Cognitive Sciences and Picower Institute for Learning and Memory, Massachusetts Institute of Technology, Cambridge, Massachusetts, United States of America; 3 Department of Molecular Medicine, Max-Planck Institute for Biochemistry, Martinsried, Germany; Cambridge University, United Kingdom

## Abstract

Binocular vision requires an exquisite matching of projections from each eye to form a cohesive representation of the visual world. Eye-specific inputs are anatomically segregated, but in register in the visual thalamus, and overlap within the binocular region of primary visual cortex. Here, we show that the transmembrane protein Ten_m3 regulates the alignment of ipsilateral and contralateral projections. It is expressed in a gradient in the developing visual pathway, which is consistently highest in regions that represent dorsal visual field. Mice that lack Ten_m3 show profound abnormalities in mapping of ipsilateral, but not contralateral, projections, and exhibit pronounced deficits when performing visually mediated behavioural tasks. It is likely that the functional deficits arise from the interocular mismatch, because they are reversed by acute monocular inactivation. We conclude that Ten_m3 plays a key regulatory role in the development of aligned binocular maps, which are required for normal vision.

## Author Summary

The visual world is represented within the brain as a series of maps of visual space. In species with binocular vision, the inputs from the two eyes are aligned to form a cohesive map; little is known about how this organisation is achieved during development. We show that a transmembrane protein, Ten_m3, plays an important role. Ten_m3 is required for the guidance of uncrossed retinal axons: uncrossed projections from the eye to the brain map aberrantly in mice that lack Ten_m3, although crossed projections map normally. Consequently, projections from the two eyes are not aligned in these mice. We show that this mismatch has devastating consequences for vision. Mice lacking Ten_m3 perform very poorly in behavioural tests of visual function. The deficits are a direct result of the mismatch, because acutely silencing inputs from one eye restores visual behaviour. This remarkable and rapid recovery suggests the mismatch of the inputs from the two eyes leads to functional suppression in the brain. We conclude that Ten_m3 acts as an eye-specific guidance cue for retinal axons and is required to produce aligned projections from the two eyes, and further, that this is critical for normal visual function.

## Abstract 3

Ten_m3, a transmembrane protein, has a newly discovered role in guiding retinal axons, aligning projections from the two eyes, and thereby mediating binocular vision.

## Introduction

The functional capabilities of the central nervous system are critically dependent on highly specific patterns of neural connectivity that are established during early development. In many parts of the brain, axonal projections form a topographic map whereby relative spatial relationships between afferent and target fields are maintained. This spatial mapping is thought to be important in maintaining the integrity of information at successive levels of processing. Molecular gradients have been shown to play a key role in establishing normal topography in the developing visual system [1–5]. Binocular vision places an additional requirement on map formation, because the projections from each eye need to be both topographic and also aligned with the projections from the other eye. Although there have been substantial advances in our understanding of the mechanisms that control whether retinal ganglion cells (RGCs) project ipsilaterally or contralaterally [6–10], the cues that allow these projections to form aligned binocular maps within their targets have remained elusive, despite attempts to identify them [11]. The only family of axonal guidance molecules with a claim on a role in eye-specific mapping is the EphA family of receptor tyrosine kinases and their ligands, the ephrinAs [12,13]. However, EphA–ephrinA interactions also play a major role in the establishment of topography of contralateral visual projections to central targets [14–18], which complicates the interpretation that these molecules have a role in eye-specific patterning. Deletion of an eye-specific mapping molecule should disrupt the projections of one eye, but not that of the other, thereby causing a mismatch between the two eyes' projections in central targets. The only genetic mutation that causes an interocular mismatch reported to date is that associated with albinism, which has been well characterised functionally in Siamese cats [19–21]. In this case, however, the mismatch is due to an aberrant decussation at the optic chiasm followed by normal retinal mapping within the target, albeit on the inappropriate side of the brain [20], rather than changes in response to guidance cues in the target itself. This has some parallels with mapping defects in the achiasmatic Belgian sheepdog [22].

We recently identified Ten_m3 in a microarray screen for molecules that are differentially expressed between visual versus nonvisual pathways [23]. The Ten_m (also known as Odz and teneurin) molecules are a recently described family of highly conserved type II transmembrane proteins of unknown function. They are the vertebrate homologs [24] of the late-acting *Drosophila* pair-rule gene *Ten_m/Odz* [25,26]. Recent work indicates potential roles for these molecules in mediating cellular interactions and adhesion [27]. Both Ten_m1 and Ten_m2 have been shown to be expressed in the developing avian visual system [28,29]. The aim of the current study was to investigate a potential role for Ten_m3 in the development of the visual pathway. We show that Ten_m3 has an important and previously unsuspected role as an eye-specific mapping molecule: the absence of Ten_m3 causes a dramatic change in the mapping of ipsilateral retinal inputs. Significantly, these occur in the absence of a major change in the mapping of geniculocortical or contralateral retinogeniculate projections. Consequently, there is a mismatch in binocular mapping, and this is associated with major deficits in the performance of visually mediated behavioural tasks. It is likely that these deficits are a result of functional suppression arising from the mismatch, because acutely silencing the inputs from one eye in adult animals restores vision.

## Results

### Expression of Ten_m3 in the Visual Pathway

**Figure 1:**
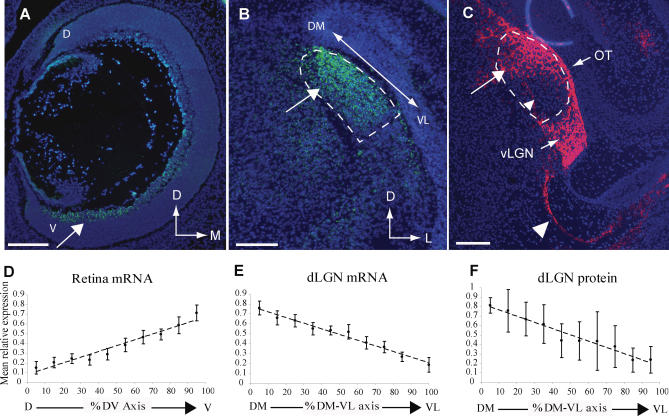
Expression of Ten_m3 in Developing Visual Pathway The expression of Ten_m3 is shown using in situ hybridisation (A) and (B) or immunohistochemistry (C). (A) Coronal section through the retina at E16. Ten_m3 is highly expressed in the retinal ganglion cell layer where it is highest in ventral retina (arrow). (B) Coronal section through the dLGN at P0. Ten_m3 is expressed high dorsally (arrow) and low ventrally. (C) Coronal section through the dLGN showing immunoreactivity for Ten_m3. Expression is highest in the dorsal region of the nucleus (arrow). High expression is also seen laterally in the optic tract (OT) and in the vLGN. Expression is also visible in fine fascicles traversing the nucleus (small arrowhead) and in larger bundles that appear to be heading to and/or from the internal capsule (large arrowhead). The dashed line delineates the boundaries of the dLGN in (B) and (C). Orientations are as indicated. D, dorsal; L, lateral; M, medial. Scale bars indicate 100 μm. (D–F) Graphs plotting relative Ten_m3 expression at P0–2. Ten_m3 mRNA is graded across the dorsoventral axis of the retina (D) and the DM-VL axis of the dLGN ([E] DM-VL axis illustrated by the double-headed arrow in [B]). Expression levels of Ten_m3 protein (F) are also graded along the DM-VL axis of the dLGN.

We first analysed the normal expression of Ten_m3 in the developing visual pathway using in situ hybridisation. Ten_m3 mRNA was expressed in the innermost layer of the developing retina, corresponding to the developing RGC layer at embryonic day (E)16 (Figure 1A). The differential expression across the dorsoventral axis of the retina was quantified using real-time polymerase chain reaction (PCR) at P0 and from sections through the retina at P2. Ten_m3 mRNA was 3.1 ± 0.50 (mean ± standard error) fold higher in samples from ventral retina compared to dorsal at P0 (*p* < 0.05, Pairwise fixed reallocation randomisation test [30]). Within the RGC layer, expression of mRNA for Ten_m3 was in a linear, high-ventral to low-dorsal gradient by P2 (Figure 1D). In situ hybridisation showed that Ten_m3 was also expressed in the dorsal lateral geniculate nucleus (dLGN), where it was highest dorsally and lowest ventrally (Figure 1B). Quantification along the long (dorsomedial to ventrolateral [DM-VL]) axis of the dLGN revealed that Ten_m3 expression is consistently in a linear gradient (Figure 1E). Interestingly, since ventral retina projects topographically to the dorsal region of the dLGN, these data show Ten_m3 is highest in corresponding regions of the visual pathway, suggesting a potential role in the establishment of retinogeniculate projections. These regions include, although they are not limited to, the sites of origin [31] and termination [32] of ipsilaterally projecting RGCs. Ten_m3 expression was also observed, though at low levels, in the ventral LGN (vLGN; n.b.: the vLGN is a distinct nucleus of the ventral thalamus as opposed to the ventral region of the dLGN). Immunostaining for Ten_m3 showed a similar pattern within the dLGN as seen with in situ hybridisation, with particularly strong reaction product in the dorsal region of the nucleus (Figure 1C; arrow, and 1F). Labelling was also seen superficially in the optic tract, which is the region from which retinal inputs enter the dLGN. Fine fascicles resembling fibres were also observed running through the dLGN and more ventrolaterally in tracts running to/from the cortex. It therefore seems likely that the antibody staining seen in the dLGN reflects the presence of Ten_m3, not only in cells of the dLGN, as shown by in situ hybridisation (Figure 1B), but also within fibre tracts that enter and leave the nucleus from the retina and cortex. This is consistent with the presence of Ten_m3 immunoreactivity in the white matter below visual cortex [23]. Quantification revealed that the high dorsal–low ventral expression pattern is also seen at the protein level (Figure 1F), although the gradient is not as smoothly linear as for mRNA. This is most likely due to presence of protein on afferent and efferent axons as well as on dLGN cells. The antibody staining also showed reaction product in the vLGN. As for the dLGN, this is likely to represent Ten_m3 expression in the vLGN neurons, as well as on retinal axons.

### Anatomical Analyses of Ten_m3 Knockout Mice

**Figure 2:**
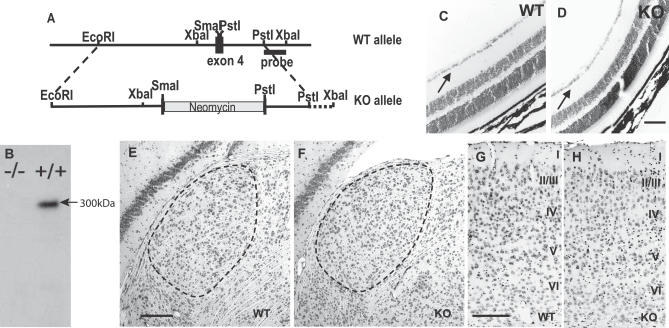
Visual Structures Have Normal Appearance in Ten_m3 KO Mice (A) Schematic diagram illustrating the strategy used to generate Ten_m3 KO mice. (B) Western blot stained with an antibody for Ten_m3. A prominent band of approximately 300 kDa corresponding to Ten_m3 is visible in the WT (+/+) mouse. This is absent in the KO(−/−) mouse. (C–H) Nissl-stained sections through retina (C) and (D), dLGN (E) and (F), and cortical area 17 (G) and (H) from WT mice (C), (E), and (G) and Ten_m3 KO mice (D), (F), and (H). No differences in size or cellular density of these structures can be discerned. Lamination of the retina and visual cortex also appears normal. The RGC layer is indicated by arrows in (C) and (D), and cortical layers are indicated with Roman numerals in (G) and (H). The borders of the dLGN are indicated by a dotted line in (E) and (F). Scale bar in (D) indicates 100 μm, which applies also to (C); scale bars in (E) and (G) indicate 200 μm, which also apply to (F) and (H), respectively.

The above data demonstrate that the transmembrane protein Ten_m3 is expressed in both afferent axons and target structures of the developing visual pathway in a gradient that is consistently highest in regions that correspond topographically. This, together with evidence from a previous study that showed that Ten_m3 promotes homophilic interactions between cells and their processes [23], suggested that this molecule may play an important role in regulating appropriate connectivity of the visual pathway. To investigate its functional role, a Ten_m3 knockout (KO) mouse was generated by disruption of exon 4, which encodes the transmembrane region of the protein (Figure 2A). Ten_m3 is an approximately 3,000–amino acid type II transmembrane protein; the transmembrane region is located at around 300 amino acids from the amino terminal [24]. Quantitative PCR showed that although Ten_m3 mRNA is present in the KO, it is significantly down-regulated (0.24 ± 0.05 fold; *p* < 0.001, Pairwise fixed reallocation randomisation test) compared to wild type (WT), suggesting that it may be targeted for degradation rather than synthesized into protein. Western blots using an antibody directed against the extracellular domain of Ten_m3 on brain lysates from WT mice showed a single band of approximately 300 kDa corresponding to the Ten_m3 protein, whereas homozygous KO mice lacked this (Figure 2B), confirming the effectiveness of the KO strategy. The presence of a severely truncated form of the protein (up to a maximum of around 10% of the normal length), corresponding to part of the intracellular domain, cannot be excluded at this stage. If present, the truncated protein would be at low levels, however, with respect to WT.

Ten_m3 homozygous KOs are viable and survive into adulthood. Their numbers from heterozygote breedings are typically less than 25%, suggesting some embryonic lethality, although the reasons for this are currently unknown. No phenotype was observed in heterozygotes. Ten_m3 KOs often appeared a little smaller than their WT littermates during early postnatal development, although no specific developmental delays were evident. By adulthood, the KO and WT mice are of a similar size and weight (WT: 21.56 ± 1.01g, *n* = 5; KO: 20.58 ± 1.26 g, *n* = 5; *p* > 0.5, *t*-test). Many adult Ten_m3 KO mice have a slightly curly tail and/or a humped back. The appearance of the brain is normal, and histological observations did not reveal any major changes in brain structure or organisation. Most importantly, no differences in size, cellular density, or lamination were apparent in Nissl-stained sections through retina, dLGN, or visual cortex of Ten_m3 KOs compared to the WT mice (Figure 2C–2H).

**Figure 3:**
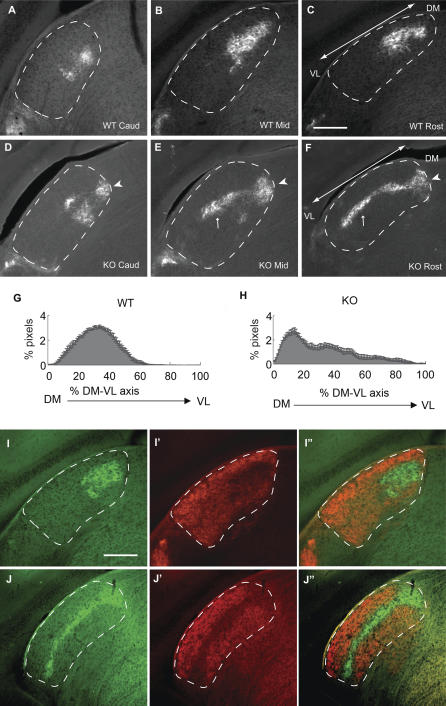
The Ipsilateral Retinogeniculate Projection Is Altered in Ten_m3 KO Mice (A–C) A caudal to rostral series of coronal sections through the dLGN of a 3.5-wk-old WT mouse showing the normal distribution of ipsilateral terminals in the dLGN following transport of fluorescent CTB from the eye. Ipsilateral projections consistently target the dorsomedial (DM) region at all rostrocaudal levels of the nucleus. (D–F) As for (A–C), but from a Ten_m3 KO mouse. Marked alterations in the targeting of ipsilateral projections are clearly visible. In caudal sections (D), the label is confined to the DM region of the nucleus similar to WTs, but, unlike WTs, the label is expanded dorsomedially such that it abuts the border of the nucleus (arrowhead). In the centre of the nucleus (E), the projection expands both towards the ventrolateral (VL) region of the nucleus (small arrow) and the dorsomedial (arrowhead). This is more obvious in the rostral region of the nucleus (F) where the VL tail approaches the border of the nucleus. Note that although the ipsilateral region is elongated in KOs, it is also narrower. The double-headed arrows indicate the DM-VL axis along which the distribution of label was quantified as shown in (G) and (H). (G and H) Plots of the mean ± standard error of the mean (s.e.m.) distribution of the ipsilateral projection along the DV-ML axis in five WT (G) and five KO (H) animals. In WTs, a smooth bell-shaped curve is seen that rises to peak around 30%–40% of the DV-ML axis and decreases to low values by around 60% of the axis. In KOs, the curve rises sharply to peak at around 10% of the DV-ML axis (DM expansion), but the tail of the distribution declines much more gradually with some label extending to 100% of the DM-VL axis (VL expansion). The distributions are significantly different (*p* < 0.0001, Kolmogorov-Smirnov test). (I–J") Relationship of ipsilateral and contralateral projections in WT (I–I") and Ten_m3 KO (J–J") mice. Coronal sections through the dLGN following intraocular injections of red or green CTB into the right or left eye, respectively. The images (I–I″) and (J–J″) are photomicrographs of the same section showing, respectively, the ipsilateral label (green), contralateral label (red), and a merged image of the two for each section. In WTs, the ipsilateral projection is confined to a patch in the dorsomedial quadrant of the dLGN (I) as above, whereas the contralateral label projection avoids this region and fills the rest of the dLGN (I′). The relationship between the ipsilateral and contralateral projections is seen more clearly in the merged image (I″). In Ten_m3 KOs, the ipsilateral projection (J) is dramatically elongated along the DM-VL axis of the dLGN as above. The contralateral projection (J′) avoids the regions occupied by the ipsilateral terminals (J″) even though they now map to regions usually occupied by contralateral terminals. The borders of the dLGN are indicated by the dashed line in (A–F) and (I–J″). Dorsal is to the top, and lateral to the left. Scale bar indicates 200 μm, which applies to all micrographs

To investigate a role for Ten_m3 in regulating the formation of visual projections, the organisation of ipsilateral and contralateral retinal projections was examined in Ten_m3 KOs during the fourth postnatal week when the projection is adult-like [33]. RGC axons from each eye were labelled with cholera toxin subunit B (CTB) conjugated to either a red or green fluorescent dye. In WTs, the ipsilateral projection consistently formed a distinct patch within the dorsomedial quadrant of the dLGN at all rostrocaudal levels of the nucleus (Figure 3A–3C). A dramatic change in the targeting of the ipsilateral projection was observed in Ten_m3 KOs. The difference was least apparent in sections though the caudal region of the nucleus, where the ipsilateral patch was confined to the dorsomedial quadrant, although unlike in WTs, the patch was expanded such that it abutted the dorsomedial border of the dLGN (Figure 3D). More rostrally, the difference was much more marked; the ipsilateral patch was narrower and elongated along the DM-VL axis of the dLGN (Figure 3E and 3F). The shape of the ipsilateral patch often appeared comet-like, with an expanded head dorsomedially and a thinner tail ventrolaterally. The change seen was symmetrical, highly consistent between animals, and maintained into adulthood (see below). In some cases, two distinct patches of label were visible in a given section, although examination of the rostrocaudal series of sections revealed that the two patches are always continuous with each other. The distribution of ipsilateral label was quantified along the DM-VL axis of the dLGN for the entire rostrocaudal extent of the nucleus in five each KOs and WTs (Figure 3G and 3H) and illustrates the highly consistent nature of the mapping change. Statistical analysis confirmed that the change is highly significant (*p* < 0.0001, Kolmogorov-Smirnov test; *n* = 5 each for WT and KO).

In WT mice, the ipsilateral and contralateral projections are anatomically segregated; contralateral projections fill all regions of the dLGN not occupied by ipsilateral terminals, including the ventrolateral region of the nucleus (Figure 3I–3I"). Given the change in the mapping of ipsilateral axons in KOs, it was of interest to determine whether ipsilateral and contralateral axons are still segregated. Comparison of the distribution of the ipsilateral and contralateral projections in any given KO (Figure 3J–3J") indicated that the terminals from the two eyes are clearly segregated from each other, despite the fact that ipsilateral axons now map to regions of the dLGN that would normally be innervated by axons from the contralateral eye. Since activity is believed to be important in segregating terminals from the two eyes in the dLGN [13,34], this suggests that at least some aspects of retinogeniculate activity are normal in Ten_m3 KOs. Analysis of the relative area occupied by ipsilateral terminals across sections showed no change in KO mice (WT: 14.5 ± 1.03%, *n* = 5; KO: 14.7% ± 1.02, *n* = 5; *p* > 0.5, *t*-test). It thus seems that the ipsilateral zone of the dLGN is elongated rather than expanded per se.

**Figure 4:**
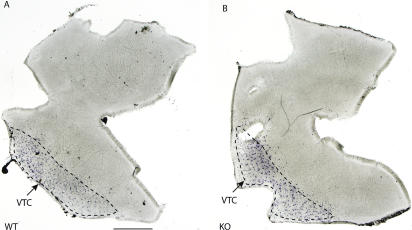
The Region of Origin of Ipsilaterally Projecting Retinal Ganglion Cells Is Not Altered in Ten_m3 KO Mice Whole-mount retinas following reaction for WGA-HRP retrogradely transported from the ipsilateral dLGN nucleus. Labelled cells are present in similar numbers (see text) within the same region of retina and the VTC in both WT (A) and KO (B) mice. Scale bar indicates1 mm, which applies to both images.

In mice, over 95% of RGCs project contralaterally. The adult ipsilateral projection arises from RGCs within the peripheral ventrotemporal retina known as the ventrotemporal crescent (VTC). Within the VTC, approximately 15% of RGCs project ipsilaterally and the remaining RGCs project contralaterally [31]. In normal mice, the ipsilaterally projecting cells map to the dorsal (binocular) region of the dLGN. More ventral regions of the dLGN exclusively receive inputs from the contralateral retina. The observed alteration in the mapping of ipsilateral projections in Ten_m3 KOs could, therefore, potentially be accounted for by two distinct mechanisms: (1) axons from dorsal or nasal retina make an inappropriate choice at the optic chiasm and project ipsilaterally, but then make an appropriate topographic choice within the target itself (crossing defect, but in reverse direction to an albino); or (2) axons make an appropriate choice at the chiasm, but make an inappropriate topographic choice within the target nucleus (mapping defect). To discriminate between these possibilities, retrograde tracing studies were performed to determine the point of origin and number of ipsilaterally projecting RGCs in Ten_m3 KOs versus WTs. Injections of wheat-germ agglutinin conjugated to horseradish peroxidase (WGA-HRP) were made into the dLGN of four adult WT and four KO mice. Retrogradely labelled cells filled the contralateral retina of both groups of mice, confirming the accuracy of the injections. No difference in the distribution of retrogradely labelled cells across the contralateral or ipsilateral retinas, including the VTC, was apparent between the groups. Most importantly, in the ipsilateral retinas, the vast majority of retrogradely labelled RGCs were located within the VTC in both WTs and KOs (Figure 4A and 4B). Quantification confirmed that there was no difference either in the size of the region containing labelled cells (WT: 1.63 ± 0.17 mm^2^, *n* = 3; KO: 1.62 ± 0.17 mm^2^, *n* = 3; *p* > 0.9, *t*-test; *n* = 3) or their density within the VTC (WT: 548 ± 127 cells/mm^2^; KO: 612 ± 92 cells/mm^2^; *p* > 0.7, *t*-test). These numbers provide an estimate of just under 1,000 ipsilaterally projecting RGCs in both WTs and KOs, similar to previously published values in pigmented mice [31]. The lack of a change in the number of ipsilaterally projecting cells is also consistent with the observation that the proportion of the dLGN occupied by ipsilateral terminals is not altered in KOs. A small number of retrogradely labelled cells were seen scattered over other regions of the ipsilateral retina in both KOs and WTs, but no difference in their occurrence was apparent. Retrograde labelling from the superior colliculus in developing animals (postnatal day [P]3–5), labelled only a subset of RGCs, but produced qualitatively similar results (unpublished results). These data present strong evidence that the change in mapping is due to a mapping defect within the dLGN and not to inappropriate crossing of cells from more dorsal or nasal retina at the optic chiasm.

**Figure 5:**
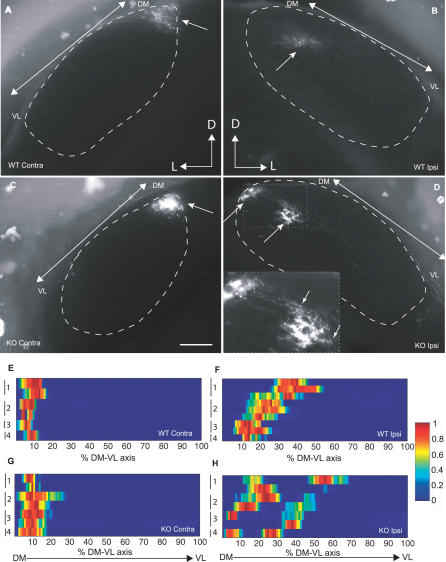
Focal Ipsilateral Terminations Are Altered in Ten_m3 KO Mice (A–D) Contralateral (A) and (C) and ipsilateral (B) and (D) coronal sections through the dLGN of WT (A) and (B) and Ten_m3 KO (C) and (D) mice following focal injections of DiI into the peripheral ventrotemporal retina. Injections were of a similar size in WTs and KOs, and resulted in a single TZ near the dorsomedial (DM) border of the contralateral dLGN in both groups of animals (arrows in [A] and [C]). Labelling in the ipsilateral dLGN from these same injections shows a different pattern in WTs (B) versus KOs (D). In WT mice, a single patch of label is in a location similar to the position of the ipsilateral patch following bulk injections in WTs (arrow in [B]; compare to Figure 3B). In the Ten_m3 KO mice, there are two distinct foci, one abutting the dorsomedial border of the dLGN and the other more ventrolateral (arrows in [D]). A higher power view of the region indicated in the dotted box is shown in the inset: a number of axons can be seen to pass directly from one TZ to the other rather than enter the more dorsomedial TZ from the optic tract. In addition, axons appear to bifurcate near the more ventrolateral TZ (small arrows) and send branches towards each TZ. The borders of the dLGN are marked with the dashed line. The arrow indicates the DM-VL axis along which the distribution of label was quantified. D, dorsal; L, lateral; M, medial. (E–H) Heat maps showing the complete distribution of terminal label in all labelled sections through contralateral (E) and (G) and ipsilateral (F) and (H) dLGN from four WT (E) and (F) and four KO mice (G) and (H). Each horizontal line represents a section. In some mice, label was observed in more than one section; sections taken from the same animal are grouped by a vertical line on the left-hand side of each heat map. Each animal is numbered. The *x*-axis plots percent distance along the DM-VL axis of the dLGN as defined above. In contralateral sections, label was always confined to the DM region of the dLGN and appears similar between WTs and KOs. A single patch of ipsilateral label is seen in ipsilateral sections from WTs. Comparison of the position of the label across sections from a given animal indicates that these correspond to a single TZ. Two distinct patches of label are seen in virtually every labelled ipsilateral section from KOs. Scale bar in (C) indicates 100 μm, and applies to all micrographs except the inset of (D), where it represents 50 μm.

To investigate the topography of retinal projections, we made focal injections of the carbocyanine dye, DiI, into the retina of P11–12 animals (this age was chosen because it is just after the period when topography becomes refined [34]). Following a focal injection of DiI into the VTC of WTs, a single, well-localised terminal zone (TZ) was consistently observed contralaterally as a densely labelled region abutting the dorsomedial border of the dLGN (Figure 5A). The ipsilateral TZ was typically larger and positioned slightly more ventrally (Figure 5B), consistent with the normal characteristics of the ipsilateral projection in mice [32,35]. In Ten_m3 KOs, a single, well-localised TZ was seen contralaterally, adjacent to the dorsomedial border of the dLGN (Figure 5C), as in WTs. In contrast, ipsilateral axons in Ten_m3 KOs consistently showed abnormalities in their targeting within the dLGN (Figure 5D). Most strikingly, focal injections into the VTC consistently resulted in two distinct TZs within the ipsilateral dLGN: one dorsomedially and the other more ventrolaterally. This was observed in all cases examined (Figure 5E–5H). In cases in which retinal axons were well labelled (as opposed to predominantly terminal labelling), axons were observed to run in a fairly straight line between the two TZs (inset in Figure 5D), suggesting that individual axons may innervate both TZs (if different axons innervated each TZ, we would expect that retinal axons would pass directly from the optic tract to the dorsomedial TZ, rather than arriving there via the more ventrolateral TZ). The possibility that individual RGC axons have multiple terminal foci is also supported by the observation that some axons appeared to branch near the more ventrolateral TZ, sending one branch into this TZ and another dorsomedially towards the other TZ (inset in Figure 5D), although analysis at higher resolution will be needed to confirm this. Occasionally, only one TZ was visible in a given section from a KO, but comparison with an adjacent section revealed two distinct patches of label (see plot for animal 3 in Figure 5H). Although label also spanned multiple sections in WTs, comparison of adjacent sections suggested that they were always part of a single TZ (Figure 5G). To gain an objective measure of the number of TZs per section, a cluster analysis was performed. This analysis determined that there was a single cluster present in 100% of contralateral sections and in ipsilateral sections from WTs (median = 1, mode = 1, mean = 1.0 ± 0). The same analysis determined that there were two clusters in almost every labelled ipsilateral section from KOs (median = 2, mode = 2, mean = 1.9 ± 0.14). This was significantly different from all other groups (*p* < 0.01, Wilcoxon rank sum test, comparing ipsilateral sections in KOs versus ipsilateral sections in WT, and contralateral sections in KO and WT). The position of the contralateral patch along the DV-ML axis was similar between WTs and KOs, supporting the suggestion that there is no major shift in topography of contralateral projections. As an additional control, focal injections into the dorsal retina were also performed. These experiments revealed no change in the topography of contralateral retinal projections from this region (Figure S1). No ipsilateral terminals were observed following injections into dorsal retina, consistent with the retrograde tracing studies demonstrating that the absence of Ten_m3 results in a mapping, rather than a decussation, defect.

The dLGN receives inputs from the retina and sends outputs to the primary visual cortex (area 17). We therefore examined the topographic relationship between visual thalamus and cortex in Ten_m3 KOs. This was of particular interest both because Ten_m3 is expressed in geniculocortical neurons and in developing visual cortex [23], and because compensatory changes in geniculocortical projections have been reported in Siamese cats [19]. For this, injections of biotinylated dextran amine were made into medial, central, and lateral regions of area 17 of adult WTs and Ten_m3 KOs. Labelling patterns correlated well with injection position in all animals (Figure S2). For example, injections into medial area 17 resulted in a patch of anterograde and retrograde label in ventral dLGN of both WTs and KOs (Figure S2A and S2B). Injections into more lateral regions of area 17 resulted in the normal topographic shift of the transported label to more dorsal regions of the dLGN for all animals (Figure S2C and S2D). The size of the labelled region following similar injections typically appeared slightly larger in KOs, although this difference was not significant (WT: 5.8 ± 1.3 × 10^5^ pixels, *n* = 9; KO: 6.7 ± 1.2 × 10^5^ pixels, *n* = 8; *p* > 0.5, *t*-test). That is, although there may be a subtle decrease in the precision of geniculocortical connectivity, the overall topography of the pathway appeared normal in Ten_m3 KOs.

**Figure 6:**
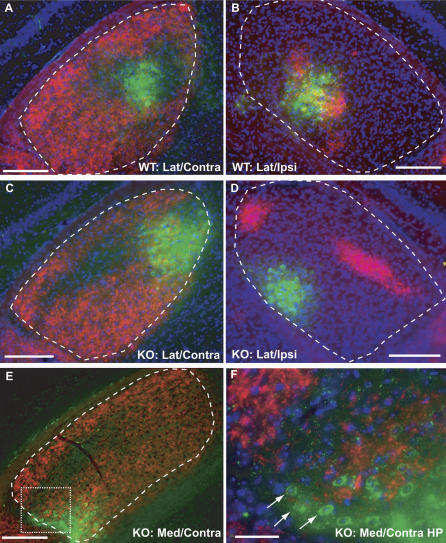
The Retinogeniculate Mismatch Is Transferred to the Cortex Coronal sections through the dLGN in adult WT (A) and (B) and KO (C–F) mice following anterograde transport of red CTB from the ipsilateral (Ipsi) (B) and (D) or contralateral (Contra) (A), (C), (E), and (F) eye and of green CTB from either the lateral (Lat) (A–D) or medial (Med) (E) and (F) region of cortical area 17. In WTs, the location of dLGN cells retrogradely labelled from the lateral (binocular) region of area 17 corresponds well with the position of ipsilateral retinal terminals (B), or with the gap in the position of the contralateral retinal terminals, which corresponds to their location (A). All retrogradely cells are located either within, or close to, the ipsilateral patch. In KOs (C) and (D), the position of cells retrogradely labelled from lateral area 17 is similar to WTs. In the dorsal region of the nucleus, this shows some overlap with the location of the ipsilateral patch. However, since the ipsilateral patch is narrower and extends much more ventrolaterally, many of the retrogradely labelled cells are located at some distance from the ipsilateral patch; no retrogradely labelled cells are seen in the ventrolateral region of the ipsilateral patch following injections into lateral area 17, suggesting that geniculocortical connections have not changed in a compensatory manner in KOs. Following injections into medial area 17 (E), retrogradely labelled cells are seen in the ventral region of the nucleus. In KOs, the retrogradely labelled cells are in close proximity to the ventral expansion of the ipsilateral patch. The boxed region is shown at higher power (Contra HP) in (F). Most significantly, retrogradely labelled dLGN neurons (some highlighted with arrows) are seen both within and beside the ipsilateral patch. Scale bars indicate 200 μm in (A–E) and 40 μm in (F).

**Figure 7:**
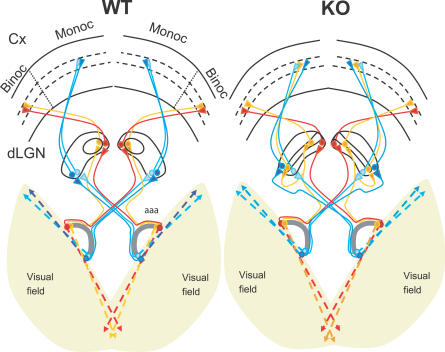
Schematic Diagram Summarising Observed Changes in Visual Circuitry in Ten_m3 KOs In WT mice (left), ipsilateral retinal fibres (orange) project exclusively to the dorsomedial dLGN. Contralaterally projecting RGCs, which view similar regions of the central, binocular region of the field (red), also project to the dorsal (binocular) region of the dLGN. Contralaterally projecting fibres from more nasal retina receive inputs from the peripheral, monocular region of the visual field and project to more ventral dLGN (blue fibres). The dLGN projects topographically to visual cortex with dorsal (binocular) dLGN represented laterally (binocular region of area 17) and ventral dLGN represented more medially (monocular region). Thus, there is a continuous representation of the contralateral visual field across the visual cortex. Note that nearby regions of visual space map to nearby regions of visual cortex, creating a single, cohesive map of the visual world. In Ten_m3 KO mice, the contralateral inputs map to dLGN similarly as described for WTs. Ipsilateral fibres map aberrantly along the DM-VL axis of the dLGN. The topographic relationship between thalamus and cortex is maintained, however. Therefore, ipsilateral inputs that project to ventral regions of the dLGN (normally the monocular segment) are transferred to more medial (normally monocular) region of area 17. Consequently, cells in area 17 that normally receive inputs only from nearby regions of the monocular visual field will now receive inputs from widely disparate regions of the monocular and binocular fields. Thus the cohesive map of visual space in the cortex is disrupted due to the mistargeting of ipsilateral projections to the dLGN. This leads to an interocular mismatch of the inputs to area 17 in Ten_m3 KOs.

In order to confirm these results with respect to the potential transfer of information from the aberrantly mapped ipsilateral eye to the visual cortex, retrograde tracing from the cortex was combined with anterograde tracing from the retina. In WTs, injections of green CTB into the lateral (binocular) zone resulted in a patch of retrogradely labelled cells in the dorsomedial region of the dLGN. Notably, all of the retrogradely labelled cells were contained either within, or immediately adjacent to, the ipsilateral patch (this was visible either as ipsilateral retinal terminals anterogradely labelled with red CTB as in Figure 6B, or with the gap in the location of labelled terminals in the contralateral dLGN as in Figure 6A). The absolute position of the cells retrogradely labelled from lateral area 17 was similar in KOs (Figure 6C and 6D); however, because the ipsilateral patch is much narrower and extends into the ventrolateral region of the nucleus, the locations of the anterograde and retrograde label did not necessarily correlate with each other. Injections into the medial (normally monocular) region of area 17 produced retrograde label in the ventral region of the dLGN in both WTs (Figure S2A) and KOs (Figure S2B). The combined anterograde and retrograde labelling confirmed that this region is in close proximity to, and overlaps with, the ipsilateral patch in KOs (Figure 6E). The presence of retrogradely labelled cells within the ipsilateral patch is clearly visible at higher power (Figure 6F). Although only seen here in low numbers, this confirms that some ipsilateral retinal inputs will be represented in medial area 17 in KOs. The small numbers most probably reflect the fact that only around 15% of geniculocortical axons represent ipsilateral inputs (as opposed to the 85% that represent contralateral inputs) and the associated difficulty of accurately targeting them without prior physiological mapping of injection sites. A correlation between the position of cells retrogradely labelled from medial area 17 and the ipsilateral patch was not seen in WTs (unpublished data). This indicates that the mismatch of ipsilateral and contralateral retinogeniculate inputs is transferred to the cortex. A schematic diagram summarizing the change in retinogeniculocortical mapping in Ten_m3 KOs is provided in Figure 7

### Behavioural Analyses of Ten_m3 Knockout Mice

**Figure 8:**
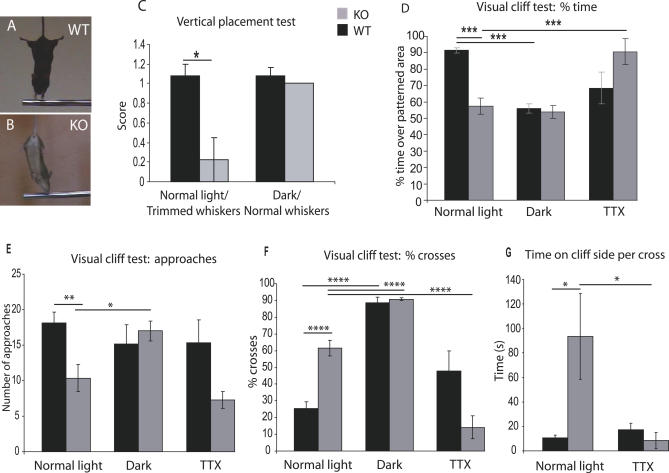
Ten_m3 KO Mice Show Deficits in the Performance of Visually-Mediated Behavioural Tasks (A–C) Vertical placement test. Examples of responses by a typical WT mouse ([A]: score of 1) and KO mouse ([B]: score of 0) are shown. WT mice scored significantly better (1.1 ± 0.11; *n* = 10) than KOs (0.22 ± 0.22; *n* = 9; *p* < 0.5, *t*-test) on this task under normal light conditions with trimmed whiskers. The ability of KO mice to perform this test using their somatosensory systems was tested by performing the test under red light (a wavelength not detected by mouse retina) with whiskers at normal length. KO and WTs performed the test equally well under these conditions. Mean scores ± s.e.m. are plotted in (C). (D–G) Results of visual cliff test (see also Videos S1, S2, and S3). (D) Total time spent in the patterned half of the box. Under normal light, WTs spent over 90% of their time in the patterned region, whereas KOs spent only half their time in this region. Under red light (dark conditions), WTs spent around half their time on each side of the cliff. There was no change in the time the KOs spent on each side of the cliff under red light. Following acute inactivation of one eye with TTX, the performance of the WT mice deteriorated, although this was not significant (*p* > 0.05, *t*-test). The TTX-treated KOs spent almost all their time on the patterned side of the box, similar to WTs under the control condition. (E–G) Visual cliff data analysed for number of approaches to the midline (E) and the percentage of approaches for which the mice crossed the midline (F) and time spent on the cliff side per cross (G). KOs approached the midline significantly less often than WTs under normal light conditions. Both groups made similar numbers of approaches to each under dark conditions. KOs made significantly more approaches in dark conditions compared to KOs under ambient light. The number of approaches made by WTs and KOs with monocular activation is similar to the number made by each group under normal light (E). Under normal light, the percent of crossings made by KOs is significantly greater than for WTs. Both groups crossed the border a significantly higher percentage of time under darkness than under normal light. The percentage of crossings is higher for WTs with TTX than for WTs under normal light conditions, though this is not significant. KOs with TTX, however, crossed the border significantly less often than KOs under normal light (F). The amount of time spent on the cliff side per cross is significantly higher for KOs under normal light conditions than for WTs under any condition or for KOs with TTX (G). A single asterisk (*) indicates *p* < 0.05; double asterisks (**) indicate *p* < 0.01; triple asterisks (***) indicate *p* < 0.001; and quadruple asterisks (****) indicate *p* < 0.0001.

We wished to determine whether the observed retinogeniculate mismatch affects vision in the Ten_m3 KO mice. Three visually mediated behavioural tasks—vertical placement, horizontal placement, and a modified version of the visual cliff test [36]—were performed under different conditions. As a control, the vertical placement test was performed under red light (a wavelength not detected by the mouse retina), and mice were graded by observers who were blind to genotype as to when they reached for the target, a metal bar, using somatosensory cues (Figure 8A and 8B) (see Materials and Methods for explanation of scores). Performance of the two groups under these conditions was identical, suggesting that Ten_m3 KOs are both capable of, and motivated to, perform the test when it is mediated by the somatosensory system (Figure 8C; WT: 1.08 ± 0.08, *n* = 6; KO 1.0 ± 0.00, *n* = 4; *p* > 0.5, *t*-test). To test the ability of Ten_m3 KOs to perform this task using the visual system, the task was repeated under normal (ambient) light with the whiskers trimmed so they could not rely on somatosensory cues. WTs scored significantly better than KOs under these conditions (Figure 8C; WT: 1.1 ± 0.1, *n* = 10; KO: 0.22 ± 0.22, *n* = 9; *p* < 0.01, *t*-test). In many cases, KOs did not reach out until their nose touched the bar (Figure 8B). The horizontal placement test was also performed, and although scores were lower for all mice, results for WTs were significantly higher than for KOs who showed no visual response to the bar at all (WT: 0.6 ± 0.18, *n* = 18; KO: 0 ± 0; *n* = 6; *p* < 0.01, *t*-test).

The visual cliff test also revealed a clear difference in the behaviour of Ten_m3 KOs (Figure 8D). For this test, animals were placed in the centre of a box with a clear acrylic (Perspex) base with a high-contrast grating appended to one half of its lower surface. The box was positioned such that the clear side protruded from the laboratory bench to give the impression of a “cliff” at the edge of the grating. The number of times animals approached the visual cliff, the percentage of these approaches that resulted in the animals crossing the cliff to the clear side of the box, the total amount of time spent in each half of the box, and mean activity levels were analysed. In the majority of cases, WTs approached the border of the grating, inspected the cliff, and then retreated to the patterned side (Video S1). In contrast, KO mice frequently walked straight over the border region and onto the cliff side without pausing (Video S2). On average, WT mice spent in excess of 90% of their time on the patterned surface (91.4 ± 1.66%; *n* = 31), whereas Ten_m3 KO mice spent approximately half their time (57.4 ± 5.0%; *n* = 18) in this region (Figure 8D). This difference is highly significant (*p* < 0.001, *t*-test) and suggests that, unlike WT mice, KOs do not exhibit a preference for the patterned half of the box. A more detailed analysis of the behaviour of the mice showed that although KOs approached the cliff less often than WTs (Figure 8E; WT: 18 ± 1.5, *n* = 33; KO: 10.4 ± 1.9, *n* = 14; *p* < 0.01, *t*-test), they crossed it significantly more frequently (Figure 8F; WT: 25.4 ± 4.1%, *n* = 33; KO: 61.5 ± 4.6% of approaches, *n* = 14; *p* < 0.0001, *t*-test). In addition, the average time spent on the cliff side of the box per crossing was almost 10-fold higher in KOs (Figure 8G; WT: 10.5 ± 2.1 s, *n* = 33; KO: 93.4 ± 35.0 s, *n* = 14; *p* < 0.05, *t*-test). As a control for possible differences in absolute activity levels, the average linear displacement of a randomly chosen subset of KOs and WTs was also determined; this did not differ between the groups of mice (WT: 251 ± 43 cm/min, *n* = 5; KO: 265 ± 21 cm/min, *n* = 5; *p* > 0.7, *t*-test ).

To determine whether the observed differences in the behaviour of WTs and KOs in the visual cliff test are mediated by visual cues, the same test was conducted under dark conditions (red light). The two groups performed almost identically to each other for all parameters examined (Figure 8D–8F; activity levels: WT: 765 ± 232 cm/min, *n* = 7; KO: 925 ± 249 cm/min, *n* = 5, *p* > 0.5, *t*-test; time on patterned side: WT: 55.9 ± 2.9%, *n* = 7; KO: 54.0 ± 3.8%, *n* = 5; *p* > 0.5; approaches: WT: 17.0 ± 2.8, *n* = 7; KO: 17.0 ±1.4, *n* = 5; *p* = 1, *t*-test; percent crossings: WT: 88.7 ± 3.4%, *n* = 7; KO: 90.8 ± 0.9%, *n* = 5; *p* > 0.5, *t*-test). When these values were compared to those for each group under normal light conditions, it was found that mean activity levels were markedly higher for both groups, although this did not reach statistical significance for either WTs or KOs (*p* > 0.05, *t*-test). The number of approaches in WTs showed no difference when compared to WTs under light conditions (*p* > 0.9, *t*-test), but KOs approached the border significantly more often in the dark compared to light conditions (Figure 8E; *p* < 0.05, *t*-test). Both groups also crossed the cliff significantly more often than under normal light (Figure 8F; *p* < 0.0001 for both groups, *t*-test). Most significantly, both groups of mice spent approximately half their time on the patterned side (Figure 8D). These values are significantly different from those obtained for WTs under ambient light (*p* < 0.001, *t*-test), but essentially identical to those for KOs under ambient light. Together, these results suggest that the marked difference in the behaviour of the WTs and KOs in the visual cliff test is indeed mediated by visual cues. They also indicate that the KOs have the capacity to distinguish light from dark, even though they show little behavioural response to the visual cliff.

We postulated that, given the relatively normal histological appearance of the visual pathway, the defect in the performance of the visual cliff test may be a direct result of the interocular mismatch in the retinogeniculocortical pathway (see Figure 7) rather than due to more generalised effects of Ten_m3 on neural connectivity. To test this hypothesis, the visual cliff test was performed on mice that had inputs from one eye acutely silenced via an intraocular injection of the sodium channel blocker tetrodotoxin (TTX). The effectiveness of the blockade was confirmed by checking that the pupillary light reflex was absent in the injected eye at the time of the test (this is normally present in Ten_m3 KOs; C. A. Leamey and A. Sawatari; unpublished data). Activity levels were not different from those obtained under normal light conditions (WT: 330.6 ± 51.3, *n* = 6; *p* > 0.2, *t*-test; KO: 273.3 ± 40.6, *n* = 7; *p* > 0.7, *t*-test), suggesting that the drug did not compromise the mobility of the mice. The number of approaches to the cliff made by WT mice with monocular TTX (Figure 8E; 15.3 ±3.2, *n* = 6) showed no difference to WT mice without TTX (*p* > 0.4, *t*-test). The proportion of times they crossed the border (Figure 8F; 47.9 ± 12.1, *n* = 6; *p* > 0.1) and total time spent over the patterned surface (Figure 8D: 68.9 ± 6.4%, *n* = 7; *p* > 0.05, *t*-test) were, however, both noticeably different. Although the differences were not statistically significant, the change in behaviour associated with monocular TTX was of concern.

To see whether the decrease in the performance levels of the WTs with TTX was a direct consequence of the loss of part of the visual field, the data were analysed to see whether the behaviour of the mice correlated with the eye that was facing the cliff as they approached the border. For example, the mice often approached the cliff either at an acute angle, or close to one of the side walls. If the active eye was facing away from the cliff, or towards the wall, the cliff would be largely or completely out of the field of view of the active eye. The number of approaches, and associated crosses, were therefore subdivided into those made while the cliff was predominantly within the field of view of the active versus the inactive eye. When the analysis criteria were restricted such that only approaches where a clear bias for one eye versus the other was apparent, we found that only a minority of these (36.3 ± 13.2%, *n* = 6), were associated with the mouse crossing the border when the cliff was predominantly within the field of view of the active eye. Expanding the criteria to include cases where the cliff was within the field of view of both of the active and inactive eye produced similar results (35.3 ± 11.9%, *n* = 6). These values are not different from the percent crosses made under normal light conditions (*p* > 0.45, *t*-test). In contrast, a very high proportion of approaches made where the active eye was facing away from the cliff were associated with a crossing (81.9 ± 8.2%, *n* = 6). This value is significantly higher than the percent crosses under normal light (*p* < 0.001, *t*-test), and the percent crosses made when approaches were made using the active eye (*p* < 0.05, *t*-test) or both eyes (*p* < 0.05, *t*-test), and is in fact similar to the percent crosses made under dark conditions. These results show that the decrease in the performance of the WTs with monocular TTX is directly associated with the loss of part of the visual field, rather than with other effects of the drug. The time spent on the cliff side of the box per cross did not show a significant difference compared to untreated WTs (Figure 8G; 17.2 ± 5.2; *p* > 0.2, *t*-test).

The behaviour of the Ten_m3 KO mice following monocular activity blockade was dramatically altered in comparison to their behaviour without TTX. Under these conditions, the KOs approached the border a similar number of times (7.3 ± 1.2, *n* = 7) to KOs without TTX (*p* > 0.1, *t*-test) and appeared to actively investigate the cliff before retreating (Video S3), a behaviour that resembled that of WTs under normal light conditions. Following TTX administration, the KOs only crossed the border in a minority of cases (14.2 ± 6.8% of approaches, *n* = 7). This value is significantly less than for KOs without TTX (*p* < 0.0001, *t*-test). The KOs with TTX also exhibited a more than 10-fold reduction in the time spent over the cliff per crossing compared to KOs without TTX (8.4 ± 6.6 s, *n* = 7; *p* < 0.05), a value similar to WTs under normal light. On average, KOs spent 90.6 ± 7.9% (*n* = 6) of time on the patterned side during monocular blockade, almost identical to the values obtained for WTs under normal conditions. The difference between this value and that obtained from the KO mice under control conditions is significant (*p* < 0.01, *t*-test). As with the WTs, the KO mice crossed the cliff much less frequently when it was in the field of view of the active versus the inactive eye (only 4.8 ± 4.8%, *n* = 7, of approaches made with the active eye facing the cliff were associated with a crossing compared to 40.3 ± 18.6%, *n* = 7, where the inactive eye was facing the cliff). To confirm that this remarkable apparent recovery of visually mediated behaviour was due to the monocular blockade rather than individual variation, two KO mice were tested both before and during the blockade. Both of these mice spent far less time on the cliff side (reductions of 41% and 22% of total time, respectively) during the monocular blockade than before the blockade.

## Discussion

Ten_m3 is a transmembrane protein [24] that promotes homophilic interactions between cells that express it [23]. We have shown here that it is expressed in both afferent and target structures of the developing visual system in gradients that are consistently highest in regions that correspond topographically to ventral retina (dorsal visual field). We have also shown that there is an expansion of ipsilateral retinal axon terminals into ventrolateral regions of the dLGN in Ten_m3 KOs. This occurs **i**n the absence of an observable change in the origin of the ipsilateral projection or the morphology and cytoarchitecture of visual structures. Together, these data suggest a role for Ten_m3 in axon guidance, possibly acting in the manner of a chemoaffinity molecule as hypothesised by Sperry [37] to attract axons from ventral retina to dorsomedial dLGN. Interestingly, rather than this being achieved by the expression of distinct ligands and their receptors, in the case of Ten_m3, our data suggest that this may be achieved by homophilic interactions between molecules expressed in corresponding gradients across the afferent and target fields. Although homophilic interactions between cell adhesion molecules have previously been postulated to play a role in the molecular matching of afferent and target regions [38–42], our data raise the possibility that they may also play a direct role in controlling the topographic mapping within these regions. It is possible that other, yet to be discovered, receptors/ligands for Ten_m3 exist and that these contribute to the role of Ten_m3 in axon guidance.

Although there are some similarities between our data supporting a role for Ten_m3 in topographic mapping and the central tenet of Sperry's chemoaffinity hypothesis [37], the observation that ipsilateral terminals were shifted, not only ventrolaterally, but also dorsomedially, suggests, however, that the role of Ten_m3 in axon guidance may be more complex than can be accounted for by monotonic chemoaffinity gradients. Recent work has provided strong evidence for attractive and repulsive forces acting to counterbalance each other in the mapping of the retina onto central targets [43–46]. It is likely that the expression of and/or response of axons to multiple attraction/repulsion cues are affected by Ten_m3. Because repulsive EphA–ephrinA interactions are reported to confine ipsilateral axons to dorsomedial dLGN [13], the ventrolateral expansion of the ipsilateral terminal region is suggestive of changes in EphA–ephrinA expression. However, as the targeting of contralateral axons is not appreciably altered, and this is known to be affected by EphA–ephrinA interactions [14,18], it is possible that other or additional mechanisms are involved in targeting of eye-specific projections.

Mice lacking the β2 subunit of the nicotinic acetylcholine receptor also have an expanded ipsilateral projection which, unlike WTs, is not segregated anatomically from the contralateral projection into a distinct, ipsilateral layer [47,48]. This may be attributable to the absence of correlated, spontaneous retinal waves during early postnatal development [49], although the absence of the receptor in the target structures may also play a role. The fact that ipsilateral and contralateral retinal axons are segregated into distinct regions in Ten_m3 KOs suggests that at least some aspects of activity are normal in Ten_m3 KOs. It also demonstrates that segregation can occur independent of the positioning of the ipsilateral terminals. This is consistent with work in ephrinA2/A3/A5 mutants that also showed that segregation can occur independently of normal topography [13].

One of the most intriguing observations made here is the change in the mapping of ipsilateral projections in the absence of a noticeable change in the topography of contralateral projections. This provides strong evidence that different guidance cues and/or receptors mediate the mapping of ipsilateral and contralateral projections. Such differential responses are clearly necessary to produce maps that are in register for the two eyes. Ten_m3 is the first molecule to be reported that clearly acts as an eye-specific guidance molecule within target nuclei. The interpretation of previous studies that have suggested that ephrinAs have a role in eye-specific mapping is complicated by the fact that they constitute topographic cues for both ipsilateral and contralateral RGCs. Thus, although removing ephrinA gradients leads to an alteration of the ipsilateral eye projection [13], similar manipulations also cause an expansion of the topographic representation of the contralateral projection [14–18]. Indeed, both ipsilateral and contralateral projections are shifted ventrolaterally in ephrinA2/A5/A3 KOs [13,18]. This is consistent with optical imaging [1] experiments in the visual cortex of ephrinA2/A5/A3 KO mice that indicate that the topographic representation of both ipsilateral and contralateral projections is expanded in a matched way. A likely means by which Ten_m3 could affect the guidance of ipsilateral, but not contralateral, RGC axons would be if it was expressed only in the ipsilaterally projecting population. The expression pattern of Ten_m3, which extends beyond the VTC, is, however, not consistent with this possibility. Differences in expression levels between contralaterally and ipsilaterally projecting RGCs and/or interactions with other molecules that are differentially expressed in ipsilaterally versus contralaterally projecting RGCs [6–10] offer potential explanations for the specificity of Ten_m3′s role in RGC guidance; these possibilities require further investigation.

Although not previously demonstrated for central targets, the differential expression of, and response to, guidance cues by ipsilateral versus contralateral axons is well established at an important intermediate guidance point for retinal axons, the optic chiasm. Recent work has shown that the EphB1 receptor plays a key role in guiding ipsilateral projections in this region [7] and that expression of the transcription factor zic2 in the developing retina defines the adult ipsilateral projection [9]. Interestingly, Ten_ms have been shown to undergo regulated intercellular proteolysis in a manner similar to Notch [50,51]. Following binding of the extracellular domain, the intracellular domains are cleaved and then translocated to the nucleus where they interact with transcription factors. Ten_m2 has been shown to interact with zic1 [50]. Although binding partners for Ten_m3 have not yet been identified, interactions with zic genes or other molecules expressed in ipsilateral axons, such as EphB1, are possible. It is therefore likely that Ten_m3 may act both directly, as an adhesion/attractant molecule, and indirectly, via transcription of other molecules, to guide ipsilateral axons within the target. Other recent work has shown that the adhesion molecule NrCAM is required for the late-born cells of the VTC to project contralaterally [8]. Given there is no evidence that contralateral projections are affected in Ten_m3 KOs, interactions with this signalling pathway, which is distinct from the EphB1 pathway [8], seem less likely.

Another major finding of the study is the profound deficit in visually mediated behaviour exhibited by Ten_m3 KOs. The findings suggest active functional suppression of inputs from one eye by the other, rather than merely the loss of stereoscopic depth perception associated with the binocular field, for the following reasons. (1) The visual cliff test employs the ventral visual field (dorsal retina), which is not part of the binocular visual field in mice [52]. (2) The performance of Ten_m3 KOs improved to a level almost identical to that of WTs in the control condition following monocular inactivation, and WTs with TTX performed the task effectively when the cliff was within the field of view of their active eye. It is surprising that a change in the targeting of the ipsilateral projection, which accounts for only 2%–3% of the total RGC population in mice [31], has such a marked effect. The ipsilateral input is greatly amplified, however, both in terms of its area of representation in dLGN [35] and its ability to drive cortical neurons [31]. The increased dLGN representation of ipsilaterally projecting cells is maintained in the Ten_m3 KOs, thus the observed changes could lead to much larger effects than suggested by consideration of RGC number alone. Despite the profound deficits that KOs exhibit when performing behavioural tasks that require patterned vision, they appear to retain an ability to discriminate light from dark; it is possible that this is mediated via subcortical visual pathways.

Based on our anatomical data, we suggest that the mistargeting of ipsilateral axons in the dLGN in Ten_m3 KOs results in a more widespread representation of ipsilateral inputs to the visual cortex rather than being confined to the lateral third of area 17 as in WTs (Figure 7). Cells that would normally receive inputs from nearby regions of the contralateral monocular visual field will, therefore, receive inputs from widely disparate regions of monocular and binocular visual fields. Thus, there will be a mismatch of inputs to the visual cortex, potentially leading to interocular suppression, as reported in visual cortex following strabismus [53,54], in the superior colliculus following monocular deprivation [55], and in Siamese cats in which, as here, an interocular mismatch in the dLGN is transferred to visual cortex [20,21]. The dramatic and rapid apparent recovery of visual function in Ten_m3 KOs following acute monocular blockade is strongly supportive of this hypothesis: the blockade allows one cortical hemisphere to receive appropriately mapped contralateral inputs without the confounding influence of aberrant ipsilateral inputs, relieving the mismatch and thus restoring visual behaviour. Of equal interest, cortical networks upstream of the dLGN seem able to correctly interpret visual input from one eye alone, after blockade of the mismatch, and enable vision in adulthood, even though these upstream structures have never received matched inputs from the two eyes during development.

## Materials and Methods

All experiments were performed on mice. Protocols were approved by the animal ethics committees of University of Sydney, Australia, Massachusetts Institute of Technology, United States of America, and/or Lund University, Sweden, and conformed to the National Institutes of Health and the National Health and Medical Research Council guidelines.

### Expression studies.

Embryos were obtained from timed matings of C57/Black6 mice. Mothers were anaesthetised with 4% isofluorane and the embryos delivered by Caesarean section and then decapitated. Neonatal mice were anaesthetised with an overdose of sodium pentobarbital and the brains removed. For PCR analysis, the retinas were subdivided and relevant regions were dissected. RNA was extracted, and quantitative real-time PCR for Ten_m3 and a control gene was performed as described in [23]. For in situ hybridisation, tissue was frozen in isopentane on dry ice. In situ hybridisation was performed using standard procedures on 15 μm–thick fresh-frozen cryostat sections using 200 bp–long dioxigenin-labelled riboprobes to sense and antisense Ten_m3 sequences. The reaction was developed using a fluoroscein-labeled TSA-plus kit (PerkinElmer, http://www.perkinelmer.com). Antibody staining was performed using a rabbit anti-Ten_m3 [56] at 1:25, followed by a biotinylated goat anti-rabbit secondary antibody, and developed with ABC (Vector Laboratories, http://www.vectorlabs.com) and a TSA plus (fluoroscein) kit.

### Generation of *Ten-m3^−/−^* mice and genotyping.

*Ten_m3^−/−^* mice were generated with a targeting construct in which the transmembrane-containing exon 4 of the *Ten_m3* gene was disrupted with a neomycin gene (Figure 2). The targeting vector was electroporated into R1 embryonic stem (ES) cells (passage 13), and four independently targeted ES cell clones were injected into C57Bl/6 blastocysts to generate germline chimeras. The chimeric founders were crossed to C57Bl/6 females to establish heterozygous *Ten-m3^+/−^* and subsequently homozygous *Ten_m3* ^−/−^ (KO) mice. Third generation backcrosses into C57/Bl6 were performed, but KO mice did not survive well on this background. Consequently, these mice were crossed with Sv129 mice, and the strain was maintained on this background. Animals are of varied pigment. Because alterations in the ipsilateral pathway of pigmented and albino mice have been reported [31], all quantitatively assessed anatomical tracing experiments reported here on the retinogeniculate pathway were performed on pigmented mice. Qualitatively similar changes in mapping were, however, observed in animals from both pigment groups. Behavioural experiments were performed on both albino and pigmented animals. Initially, data were analysed separately for pigmented and albino animals, but because no differences in visual behaviour relating to pigment were detected, KO and WT data were pooled across pigment groups. Genotyping was performed by Southern blot or PCR using DNA isolated from tail biopsies. For this, expression lysates from the brain of WT and KO mice were reduced, resolved in 6% SDS-PAGE gels, and then transferred to PVDF membranes and incubated with an affinity-purified rabbit anti-mouse Ten_m3 antibody [56].

### Tracing studies.

All surgical manipulations were performed under anaesthesia induced and maintained by inhalation of 2%–4% isofluorane in oxygen. Intraocular injections of 0.5–1 μl of 1% CTB conjugated to Alexa Fluor 594 (red) or Alexa Fluor 488 (green) were made into the right and left eyes of Ten_m3 KO and WT littermates at P21–23. For combined anterograde and retrograde labelling, injections of green CTB were made into subregions of visual cortex and red CTB was injected into one eye as above. Animals were euthanised 1–3 d later and perfused with 0.9% saline followed by 4% paraformaldehyde in 0.1 M phosphate buffer. Coronal sections, 60 μm thick, were cut using a freezing microtome. Focal injections of a 10% solution of 1,1′-dioctadecyl-3,3,3′,3′-tetramethylindocarbocyanine perchlorate (DiI) in dimethyl formamide were made in peripheral VT retina of P11–12 mice under isofluorane anaesthesia; animals were perfused 1–2 d later. This age was chosen because topography is largely adult-like by this stage [45]. Coronal sections, 100 μm thick, were cut on a vibratome. Injections of 10% biotinylated dextran amine (BDA) were made into subregions of primary visual cortex (area 17) of adult mice using stereotaxic coordinates. Label was allowed to transport for 7–10 d before animals were euthanised, perfused as above, and reacted as described in [57]. For retrograde labelling from the thalamus, 5% WGA-HRP was injected into the dLGN using stereotaxic coordinates. Animals were perfused 16–20 h later with 0.9% saline followed by 2% paraformaldehyde in 0.1 M phosphate buffer. Retinas were dissected out and reacted as whole mounts. Coronal sections, 80–100 μm thick, were cut through the dLGN using a vibratome, and a tetramethyl-benzidine reaction was performed using standard techniques.

### Image analysis.

Images were analysed using Image J (NIH). The freehand selection tool was used to outline the perimeter of the dLGN, and the area contained within this region in arbitrary units was determined. Analyses were performed across the entire rostrocaudal extent of the nucleus. Care was taken to exclude the optic tract, intergeniculate leaflet, and vLGN from this analysis. To isolate ipsilateral projections, background was subtracted using the rolling ball function, and the area containing the ipsilateral projections was selected using the wand tool. To determine shifts along the DM-VL axis, the image was cropped at the borders of the dLGN, thresholded as above, and the coordinates of each non-zero pixel were written to a file along with information on the size of the image in arbitrary units. The DM-VL axis was divided into 100 bins, and the proportion of label in each bin, expressed as a percentage of the total ipsilateral label contained within each bin, was plotted using Matlab. Means and standard errors for five KOs and five WTs were calculated and plotted. For the analysis of focal injections, all sections containing label from four KO and four WT animals were used. Thresholded data were binned as above, and Matlab was used to generate a heat map of the distribution of label. For an objective analysis of patch number, the Image analysis toolbox (Matlab) was used to calculate the number of clusters of label in images from KO and WT animals from the thresholded images. The area of labelled regions in BDA-injected animals was calculated by thresholding all labelled images in ImageJ. Labelled pixels were written to a text file and summed across all labelled sections for each animal. Means and standard errors are presented unless otherwise stated. For determination of the area of retina containing a high proportion of ipsilaterally projecting RGCs, this region was outlined in low-power photomicrographs of retinal whole mounts using ImageJ. For estimates of cell density, two fields of view within the region containing ipsilaterally projecting RGCs were photographed using the 20× objective for three KO and three WT cases. Images were thresholded to isolate individual RGCs. The number of thresholded patches was calculated using ImageJ software.

### Behaviour.

For the vertical placement test, mice were held by the tail and lowered towards a horizontal metal bar. Scores were given by two independent observers who were blind to genotype, according to when mice made a clear reaching motion for the bar with their forepaws. A score of 2 was given for mice that reached more than 1 cm from the bar and a score of 1 for mice that reached within 1 cm, but before their nose touched the bar. A score of 0 was given for mice that touched the bar with the nose before reaching for the bar. The vertical placement test was performed under 2 conditions: normal (ambient 55Cm^−2^) light with whiskers trimmed and under red light with whiskers at normal length. For the horizontal placement test, mice were moved horizontally toward the bar and scored as above.

For the visual cliff test, mice were placed in a 60-cm × 60-cm box with a clear acrylic (Perspex) base. A high-contrast grating was attached to the underside of one half of the box. The box placed such that the clear half of the base protruded from the laboratory bench, revealing a drop to the floor of approximately 90 cm. Mice were placed in the centre of the box and their behaviour monitored for 10 min via a digital video camera mounted 1 m above the floor of the box. The data were analysed, blind to genotype, from video recordings. An approach was defined as moving from the patterned region towards the “cliff” such that the nose was within 5 cm of the midline. Crossing was defined as the animal completely crossing the midline from the patterned to the clear half of the box. Retreating was defined as moving such that the head was within 5 cm of the midline and retreating without the entire body crossing the border. Subsequent approaches were not scored until the mouse had moved outside the 5-cm range. To confirm the role of the visual system in this task, the test was also performed under dark (red light) conditions. The effect of monocular blockade of activity was determined by injecting 0.5 μl of 1 mM tetrodotoxin into the left eye under isofluorane anaesthesia as above. Some mice appeared lethargic for a few hours postinjection and so were allowed to recover from any systemic effects of the procedure for 2–16 h before testing to ensure that this would not bias the results. Activity levels were monitored to confirm this was the case by calculating the movement of the mice for 1 min. For this, a scaled grid was placed over the video monitor, and the points at which the mouse crossed the grid were plotted and measured. In addition to the other analyses performed, the approaches and crosses during monocular inactivation were subdivided into cases where the mouse approached the cliff such that it was predominantly in the visual field of the active versus the inactive eye. Approaches to the cliff that were made such that the mouse was parallel to and within 10 cm of a side wall, and thus the cliff was largely out of the field of view of the eye facing towards the wall, were included in this analysis. Approaches made at an angle such that the cliff was predominantly within the field of view of one eye rather than the other were also included. Approaches where there was no clear bias for one eye versus the other were scored separately. The effectiveness of the blockade at the time of the test was confirmed by checking that the pupillary light reflex was absent in the injected eye.

## Supporting Information

Figure S1 Projections from Dorsal Retina Are Not Altered in Ten_m3 KOs Sections through the dLGN following a focal injection of DiI into the dorsal retina in a WT (A) and a KO (B) mouse. Terminals are seen in ventral dLGN in both cases. Scale bar indicates 100 μm.
(1.3 MB TIF)

Figure S2 Connectivity between dLGN and Cortex Is Largely Normal in Ten_m3 KOs Coronal sections through the dLGN following injections of the neuronal tracer BDA into medial (A) and (B) or lateral (C) and (D) area 17 in WTs (A) and (C) and KOs (B) and (D). A dense patch of label containing retrogradely labelled cells and anterogradely labelled corticothalamic fibres (arrows) is seen in the dLGN (borders indicated by dashed line). The positions of the labelled regions are similar following injections into a particular part of the visual cortex: medial injections label ventral dLGN (A) and (B), whereas lateral injections label dorsal dLGN (C) and (D). The labelled area appears slightly larger in Ten_m3 KOs, but this difference was not significant (*p* > 0.5, *t*-test; *n* = 9 for WT and *n* = 8 for KO). Scale bar in (D) indicates 200 μm, which applies to all images.
(5.1 MB PDF)

Video S1 Example of Behaviour of Typical WT Mouse while Performing Visual Cliff Test under Normal Conditions The mouse is allowed to move freely around a box with a clear acrylic base. A contrast grating is appended below half of the box. The animal pauses when it reaches the border between the patterned and nonpatterned halves, appears to inspect the border region but does not cross over, then retreats to the patterned region.
(1.2 MB MPG)

Video S2 Example of Behaviour of Typical KO Mouse while Performing Visual Cliff Test under Normal Conditions Unlike the WT mouse, when the KO mouse approaches the border region, it crosses onto the nonpatterned side with no hesitation.
(1.1 MB MPG)

Video S3 Example Showing Behaviour of Typical KO Mouse while Performing Visual Cliff Test during Acute Monocular Inactivation Under these conditions, the KO mouse appears to inspect the border region and retreats to the patterned side. This behaviour is very similar to that observed in WT mice under normal conditions (see Video S1).
(7.7 MB AVI)

### Accession Numbers

The National Center for Biotechnology Information (NCBI) (http://www.ncbi.nlm.nih.gov) accession number for Ten_m3 is NM_011857.

## Abbreviations

CTB: cholera toxin subunit B
dLGN: dorsal lateral geniculate nucleus
DM-VL: dorsomedial to ventrolateral
E: embryonic day
KO: knockout
P: postnatal day
RGC: retinal ganglion cell
TTX: tetrodotoxin
TZ: terminal zone
vLGN: ventral lateral geniculate nucleus
VTC: ventrotemporal crescent
WT: wild type
