# Supplementary material for: Ten_m3 Regulates Eye-Specific Patterning in the Mammalian Visual Pathway and Is Required for Binocular Vision
Source: PLoS Biol. 2007 Sep 4;5(9):e241. doi: 10.1371/journal.pbio.0050241 (PMC1964777; doi:10.1371/journal.pbio.0050241)
Supplement: Supplementary file 1 [file pbio.0050241.sg002.pdf]

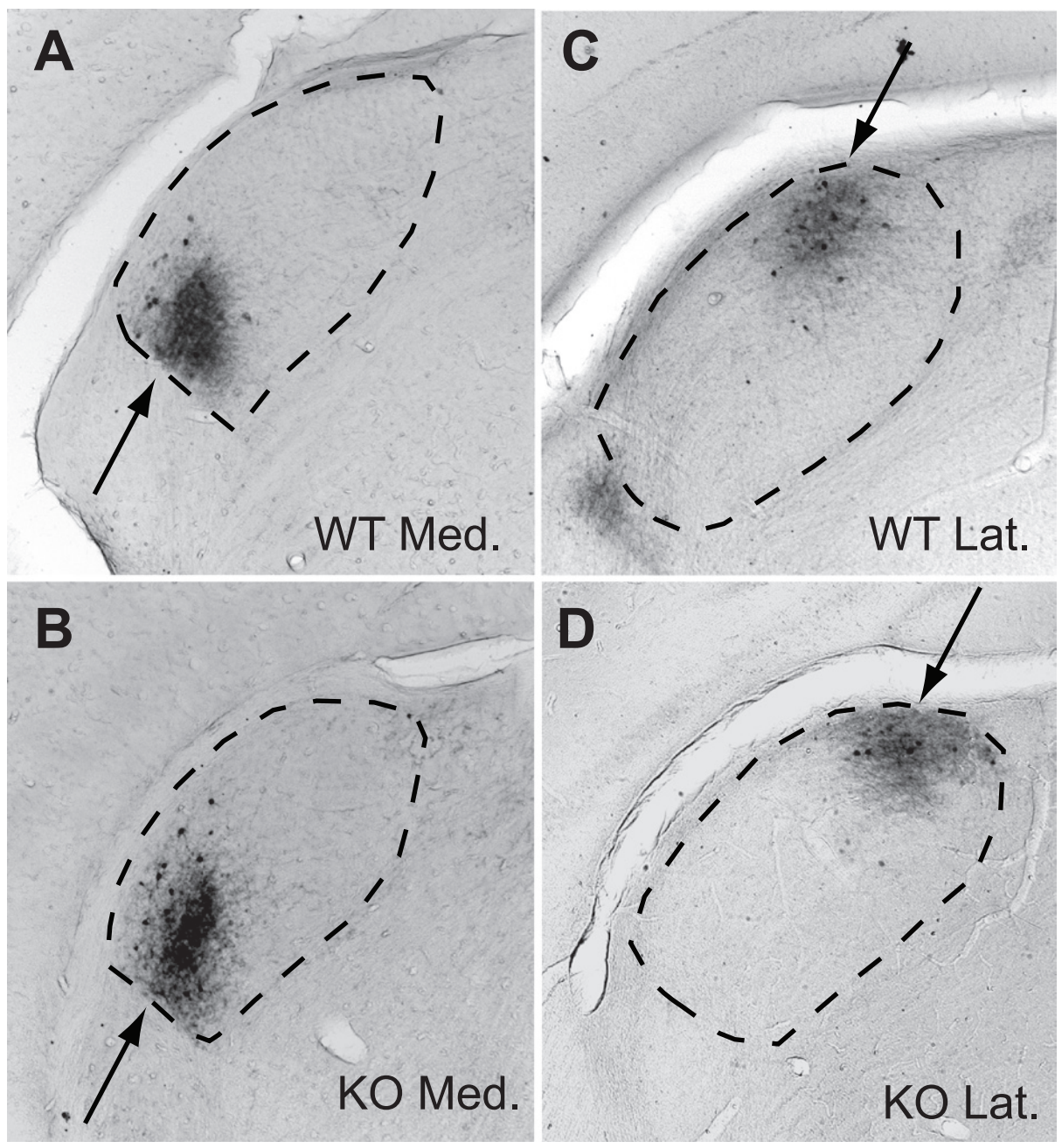

A
WT Med.
B
KO Med.
C
WT Lat.
D
KO Lat.
